# Development of a Quadruplex RT-qPCR Assay for Rapid Detection and Differentiation of PRRSV-2 and Its Predominant Genetic Sublineages in China

**DOI:** 10.3390/v17060853

**Published:** 2025-06-16

**Authors:** Guishan Ye, Siyu Xiong, Zhipeng Su, Guosheng Chen, Siyuan Liu, Zixuan Wang, Huanchun Chen, Anding Zhang

**Affiliations:** 1 National Key Laboratory of Agricultural Microbiology, Hubei Hongshan Laboratory, College of Veterinary Medicine, Huazhong Agricultural University, Wuhan 430070, China; 2 Key Laboratory of Preventive Veterinary Medicine in Hubei Province, The Cooperative Innovation Center for Sustainable Pig Production, Wuhan 430070, China; 3 Dekon Food and Agriculture Group, Chengdu 610200, China; 4 Key Laboratory of Development of Veterinary Diagnostic Products, Ministry of Agriculture of the People’s Republic of China, Wuhan 430070, China; 5 International Research Center for Animal Disease, Ministry of Science and Technology of the People’s Republic of China, Wuhan 430070, China; 6 Guangdong Provincial Key Laboratory of Research on the Technology of Pig-Breeding and Pig-Disease Prevention, Guangzhou 510000, China

**Keywords:** PRRSV, quadruplex RT-qPCR, lineage differentiation

## Abstract

Background: Porcine Reproductive and Respiratory Syndrome (PRRS) is a highly contagious disease characterized by reproductive failure in sows and severe respiratory disorders across all swine ages, causing significant economic losses. In China, the PRRSV epidemiological landscape is complex, with the coexistence of multiple lineages and frequent recombination. The major circulating strains include sublineages 1.8 (NADC30-like PRRSV) and 1.5 (NADC34-like PRRSV), along with lineages 8 (HP-like PRRSV) and 5 (VR2332-like PRRSV), highlighting the urgent need for rapid detection and lineage differentiation. Methods: A quadruplex RT-qPCR assay was developed targeting lineage-specific deletions in the NSP2 gene to simultaneously detect PRRSV-2 and differentiate NADC30-like PRRSV, HP-like PRRSV, and NADC34-like PRRSV strains. The assay was optimized with respect to reaction conditions, including annealing temperature, primers, and probe concentrations. The method’s performance was evaluated in terms of specificity, sensitivity, repeatability, stability, limit of detection (LOD), and consistency with sequencing results. Results: The assay demonstrated high sensitivity (LOD of 3 copies/μL), high specificity, and good repeatability (coefficient of variation < 1.5%). Field application using 938 samples from Guangxi A and B farms revealed NADC30-like PRRSV wild-type strains at positivity rates of 13.44% and 3.53%, respectively. Positive samples selected for sequencing were further confirmed using ORF5-based phylogenetic analysis and NSP2 deletion pattern comparison, which aligned with RT-qPCR detection results. Field application primarily detected NADC30-like PRRSV, while further validation is still needed for HP-like and NADC34-like strains. The developed quadruplex RT-qPCR assay enables rapid and simultaneous detection of PRRSV-2 and differentiation of three major lineages, providing a sensitive, specific, and reliable tool for distinguishing vaccine-derived from circulating strains and supporting targeted disease surveillance and control in swine farms.

## 1. Introduction

Porcine Reproductive and Respiratory Syndrome Virus (PRRSV), a positive-sense single-stranded RNA virus with high transmissibility, remains a major challenge to the global swine industry [1]. PRRS is characterized by reproductive failure in sows, including a return to estrus, abortion, and stillbirths, as well as respiratory disorders in piglets, such as fever, cough, dyspnea, and growth retardation [2,3]. Boars infected with PRRSV have been shown to experience testicular damage and reduced semen quality [4]. PRRSV exhibits significant immunosuppressive effects, primarily by infecting pulmonary alveolar macrophages and suppressing the host’s immune responses. This immune impairment increases susceptibility to secondary infections and contributes to persistent viral presence [5]. The virus often co-infects with Porcine Circovirus Type 2 (PCV2), which significantly exacerbates clinical manifestations, including severe respiratory distress [6]. These co-infections complicate disease management and heighten the challenges in controlling both clinical symptoms and associated costs. Economically, as reported by Holtkamp et al., PRRS alone causes annual economic losses exceeding USD 664 million in the United States [7]. In China, Zhang et al. demonstrated that PRRSV outbreaks result in economic losses of approximately CNY 1424.37 per sow during the outbreak-to-recovery phase, mainly due to reduced weaned piglet numbers and increased production costs [8]. Therefore, continuous surveillance of PRRSV prevalence is necessary to develop scientifically effective control and eradication strategies, thus reducing its economic impact and health threats to the porcine industry.

PRRSV is genetically classified into two distinct genotypes: PRRSV-1 (European type) and PRRSV-2 (North American type), with PRRSV-2 predominating in China. A recent phylogenetic analysis based on 82,237 global ORF5 gene sequences updated the classification of PRRSV-2 into 11 genetic lineages (L1–L11) and 21 sublineages. The major circulating lineages in China currently include sublineages 1.8 (NADC30-like PRRSV) and 1.5 (NADC34-like PRRSV), as well as lineages 8 (HP-like PRRSV) and 5 (VR2332-like PRRSV), forming a complex co-circulation of multiple lineages.

The prevalence of PRRSV-2 in China can be traced to the first domestic isolation of the CH-1a strain in 1995. In 2006, a highly pathogenic PRRSV strain belonging to lineage 8 emerged in China, characterized by a discontinuous 30-amino acid deletion (aa481 and aa532–560) in the NSP2 coding region and associated with widespread outbreaks marked by increased reproductive failure in sows and elevated piglet mortality [9]. The NADC30 strain was first identified in the United States in 2008 [10]. In 2013, NADC30-like PRRSV strains harboring a 131-amino acid deletion in the NSP2 region (aa323–433, aa483, and aa504–522) were introduced into China, leading to new outbreaks [11]. NADC30-like PRRSV strains have been reported to exhibit moderate pathogenicity in piglets [12]. In 2014, a novel PRRSV-2 variant, RFLP 1-7-4 IA/2014/NADC34, was reported in the United States, featuring a continuous 100-amino acid deletion (aa328–427) in NSP2 [13]. Subsequently, in 2017, the first NADC34-like PRRSV strains in China (LNWK96 and LNWK130) were isolated and confirmed to be recombinants derived from IA/2014/NADC34, ISU30, and NADC30 strains [14]. Clinical studies have shown that infections with NADC34-like PRRSV strains can result in abortion rates of 20–30% in sows [15].

These lineage-specific deletions in the NSP2 gene provide distinct molecular markers. In contrast to the ORF5 gene, which is widely used for phylogenetic analysis but lacks sufficient distinct and stable molecular signatures for rapid and accurate subtyping in-field diagnostics, molecular diagnostics based on the NSP2 variation have become increasingly valuable for practical diagnostic differentiation among PRRSV-2 lineages.

Although PRRSV-2 remains the dominant genotype in China, the prevalence of PRRSV-1 has been increasing. To date, PRRSV-1 has been identified in at least 24 regions across the country [16]. Previous studies have shown that PRRSV-1 strains isolated in China typically exhibit mild clinical symptoms and low pathogenicity in experimental infection models [17,18]. However, the emergence of certain variants, such as 181187-2, ZD-1, and AHEU2024-2671, suggests a slight increase in the virulence of some domestic strains [19,20,21]. These findings highlight the need for ongoing surveillance of both PRRSV genotypes and emphasize the importance of combining molecular and epidemiological strategies for disease control in swine production systems. Despite the widespread application of both live-attenuated and inactivated vaccines for PRRSV containment, their cross-protective effectiveness against heterologous strains remains limited. Consequently, establishing a rapid, highly sensitive strain typing method is essential to early detection and guided vaccine selection targeting circulating PRRSV variants.

The PRRSV detection techniques used to distinguish different strain types include virus isolation, RT-PCR, RT-qPCR, LAMP, RPA, dPCR, etc. [22,23,24,25,26,27]. Traditional virus isolation is considered the gold standard method for pathogen identification. However, this technique is complex, time-consuming, and unsuitable for large-scale screening or rapid diagnosis in pig farms [28]. In contrast, RT-PCR significantly reduces diagnostic time but still has limitations, as traditional methods often rely on open-tube electrophoresis, which may cause false positives due to amplicon contamination. RT-qPCR overcomes these drawbacks by monitoring the amplification process in real-time using fluorescent signals, offering high sensitivity and specificity. It enables the accurate detection of low viral loads, reduces contamination risk, and quantifies viral copy numbers based on standard curves [29], making it the most widely adopted method for PRRSV detection. Furthermore, multiplex RT-qPCR permits the simultaneous identification of multiple strains within a single reaction.

To address the clinical need for the rapid and efficient detection of PRRSV-2 and the differentiation of its predominant genetic sublineages, this study developed a quadruplex RT-qPCR assay targeting specific NSP2 deletions found in three major PRRSV lineages circulating in China: NADC30-like PRRSV, HP-like PRRSV, and NADC34-like PRRSV. This method lays a technological foundation for monitoring both vaccine strains and circulating strains in swine herds.

## 2. Materials and Methods

### 2.1. Virus

PRRSV, PEDV, PoRV A, PDCoV, PRV, CSFV, and PPV were preserved in our laboratory. (Wuhan, China).

### 2.2. Major Software Used

Primer design tool: Primer 5; Sequence alignment tools: MAFFT7.0, MEGA 11.0, and DNAMAN9.0; Visualization tool: TVBOT (https://chiplot.online/tvbot.html, accessed on 30 May 2025) [30].

### 2.3. Primer and Probe Development

We obtained 941 full-length PRRSV genome sequences from the NCBI GenBank database. Sequence alignment was performed using MAFFT, and classification was based on NSP2 gene deletions that distinguish NADC30-like PRRSV, NADC34-like PRRSV, and HP-like PRRSV strains from classical PRRSV strains. Based on these molecular signatures, we screened 941 NSP2 sequences, including 227 NADC30-like PRRSV, 337 HP-PRRSV, and 132 NADC34-like PRRSV strains. Conserved and specific regions were selected for primer and probe design with considerations of sequence alignment, mismatch tolerance, and optimal melting temperature (Tm). All candidate primers and probes were validated via BLAST (https://blast.ncbi.nlm.nih.gov/Blast.cgi, accessed on 30 May 2025) to confirm coverage of known PRRSV-2 subtypes and to avoid non-specific amplification (Table 1). The primer and probe sequences used for PRRSV-2 detection were designed by the national standard GB/T 18090-2023 Diagnostic Methods for PRRSV [31].

### 2.4. Synthesis of Standard Plasmids

The synthesized sequences of the standard plasmids are presented in Table S1.

### 2.5. Reaction Condition Optimization

Three annealing temperatures (56 °C, 58 °C, and 60 °C) were selected based on the Tm values of the synthesized primers and probes. A standard PRRSV-POS plasmid (3 × 10⁴ copies/μL) served as the positive control, while nuclease-free water was used as the negative control, with three technical replicates per temperature. RT-qPCR reactions were performed using the HiScript III U^+^ One Step qRT-PCR Probe Kit (Q225, Vazyme, Nanjing, China) according to the manufacturer’s instructions. Primer and probe concentrations were optimized within the recommended range provided in the kit manual by testing three combinations: 0.1/0.15 μmol/L, 0.2/0.3 μmol/L, and 0.3/0.45 μmol/L. Amplification was performed on an MA-688 Real-Time Quantitative Thermal Cycler (Suzhou Molarray Co., Ltd., Suzhou, China). The reaction composition is detailed in Table S2. RT-qPCR reaction program in Table S3.

### 2.6. Establishment of the Standard Curve

The concentration of the PRRSV-POS standard plasmid was quantified using a Qubit 2.0 Fluorometer (Thermo Fisher Scientific, Waltham, USA). The plasmid was then subjected to tenfold serial dilutions, producing a concentration gradient from 3 × 10^6^ to 3 copies/μL. RT-qPCR amplification was performed under optimized reaction conditions using the PRRSV-POS standard plasmid at each dilution level, with three technical replicates per dilution. A standard curve was generated by plotting the log₁₀ of plasmid copy numbers against the corresponding Ct values.

### 2.7. Sensitivity Test

The PRRSV-POS standard plasmid was subjected to tenfold serial dilutions, producing a concentration gradient from 3 × 10^6^ to 3 copies/μL. RT-qPCR amplification was performed under the optimized reaction conditions using each dilution, with three replicates per concentration. The limit of detection (LOD) for PRRSV-2, NADC30-like PRRSV, HP-like PRRSV, and NADC34-like PRRSV strains was determined as the lowest plasmid concentration at which all three replicates consistently produced positive amplification signals under the optimized reaction conditions.

### 2.8. Specificity Test

Nucleic acids (DNA or RNA) from other clinically common pathogens with similar symptoms and a high likelihood of co-infection were used as templates, including PEDV, PoRVA, PDCoV, PRV, CSFV, and PPV. Nucleic acids from C-PRRSV, NADC30-like PRRSV, and HP-like PRRSV strains were used as positive controls, while nuclease-free H₂O was used as the negative control. RT-qPCR was carried out using previously optimized conditions.

### 2.9. Repeatability Test

PRRSV-POS plasmids at concentrations of 3 × 10^5^, 3 × 10^4^, and 3 × 10^3^ copies/μL were used as templates to assess intra-assay repeatability under identical conditions. After a one-week interval, the same concentrations were used to assess inter-assay repeatability. The CV was calculated based on the obtained Ct values, and statistical analysis was subsequently conducted.

### 2.10. Concordance Analysis Between RT-qPCR Results and Sequencing Results

A total of 389 clinical samples were tested in parallel using the quadruplex RT-qPCR assay developed in this study and the national standard reference method for PRRSV detection. Among these samples, 37 tested positive, and 352 tested negative by both methods. Of the positive samples, 26 PRRSV-positive clinical samples, including blood, umbilical cord, and lung tissues, were selected for subtyping validation. Total nucleic acids were extracted and reverse transcribed, and NSP2 gene regions were amplified using primers listed in Table S4 and sequenced using standard Sanger methods.

### 2.11. Clinical Application

From January to November 2024, 938 samples were analyzed using the assay developed in this research. The collected samples were obtained from breeding sow farms A and B, located in the Guangxi Zhuang Autonomous Region, China, and included testicular fluid from boars, serum from piglets, abortion tissue from sows, as well as nasal and throat swabs. From Farm A, a total of 372 clinical samples were obtained. The PRRSV-2 positivity rate was 21.50%, and the detection rate for NADC30-like PRRSV strains was 13.44%. From Farm B, 566 samples were collected, yielding a PRRSV-2 positivity rate of 21.90% and a detection rate of 3.53% for NADC30-like PRRSV strains.

### 2.12. Statistical Analysis

Experimental data were expressed as mean ± standard deviation (SD) and analyzed using GraphPad Prism software 9.0. Differences between groups were evaluated by one-way analysis of variance (one-way ANOVA). A *p*-value of less than 0.05 was considered statistically significant, and *p* < 0.01 was considered highly significant.

## 3. Results

### 3.1. Primer and Probe Design

**Figure 1:**
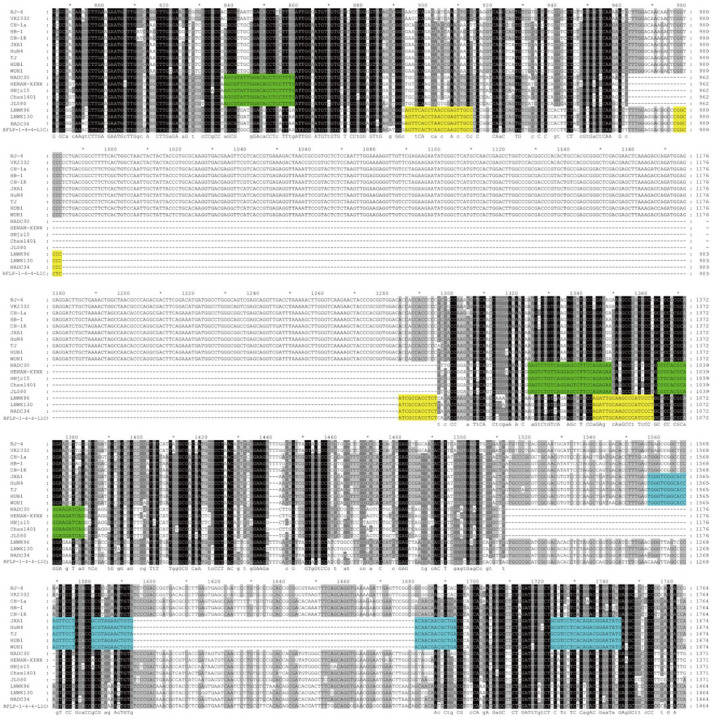
Schematic diagram of primer and probe locations. The primers and probes targeting NADC30-like PRRSV, NADC34-like PRRSV, and HP-like PRRSV are highlighted in green, yellow, and blue, respectively. * indicates a scale marker at 10 bp intervals.

Based on the molecular characteristics of NSP2 gene deletions observed in prevalent PRRSV strains in China, including NADC30-like PRRSV, NADC34-like PRRSV, and HP-like PRRSV, specific primers and probes were designed. The corresponding target regions are illustrated in Figure 1. The primer and probe sequences used for PRRSV-2 detection follow the GB/T 18090-2023 diagnostic method for PRRSV.

### 3.2. Optimization of Annealing Temperature

**Figure 2:**
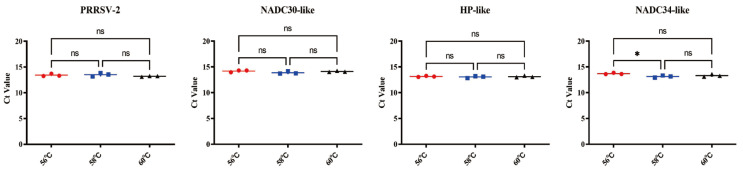
Optimization of the annealing temperature. * indicates *p* < 0.05, “ns” indicates no statistically significant difference (*p* > 0.05).

To determine the optimal annealing temperatures, a standard PRRSV-POS plasmid at a concentration of 3 × 10^4^ copies/μL was used as the template. Single RT-qPCR amplifications targeting PRRSV-2, NADC30-like, HP-like, and NADC34-like strains were conducted at 56 °C, 58 °C, and 60 °C. The results indicated that under single-target amplification conditions, no significant differences in the Ct values were observed for the PRRSV-2, NADC30-like PRRSV, and HP-like PRRSV across the tested temperatures. In contrast, the NADC34-like PRRSV target showed sensitivity to temperature: its average Ct value was slightly higher at 56 °C compared to 58 °C, and no significant differences were observed in the Ct values between the 58 °C and 60 °C groups, nor between the 56 °C and 60 °C groups (Figure 2). Given that 56 °C supported stable amplification requirements for the other three targets and ensured the compatibility of the probe fluorescence channels in the multiplex system, 56 °C was ultimately chosen as the optimal annealing temperature for the quadruplex RT-qPCR system.

### 3.3. Optimization of Primer and Probe Concentration

**Figure 3:**
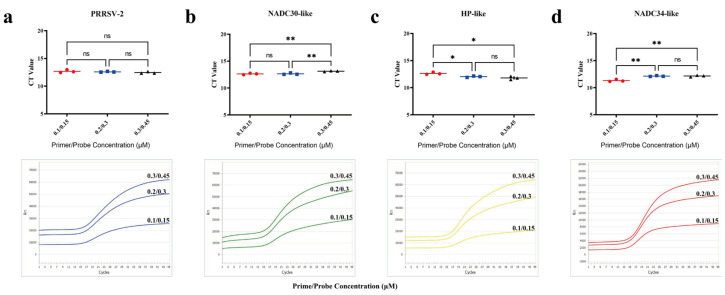
Optimization of primer and probe concentrations. (**a**–**d**) show the Ct value statistics and fluorescence amplification curves at different primer/probe concentrations for (**a**) PRRSV-2, (**b**) NADC30-like, (**c**) HP-like, and (**d**) NADC34-like, respectively. Primer/probe concentrations tested were 0.1/0.15 µM, 0.2/0.3 µM, and 0.3/0.45 µM. * indicates *p* < 0.05, ** indicates *p* < 0.01, “ns” indicates no statistically significant difference (*p* > 0.05).

To determine the optimal primer and probe concentrations, this study used the PRRSV-POS standard plasmid with a template concentration of 3 × 10^4^ copies/μL. Single RT-qPCR amplification was conducted for PRRSV-2, NADC30-like PRRSV, HP-like PRRSV, and NADC34-like PRRSV targets at primer/probe concentrations of 0.1/0.15 μmol/L, 0.2/0.3 μmol/L, and 0.3/0.45 μmol/L. The results showed that the Ct values for the PRRSV-2 target did not differ significantly between the various concentration gradients. In contrast, the HP-like PRRSV target showed a significantly lower average Ct value at the highest primer/probe concentration (0.3/0.45 μmol/L) compared to the lowest concentration (0.1/0.15 μmol/L), indicating that higher concentrations improved target capture efficiency. Notably, the NADC30-like PRRSV and NADC34-like PRRSV targets exhibited a distinct concentration-dependent effect. Although the Ct values in the 0.3/0.45 μmol/L group were significantly higher compared to the 0.1/0.15 μmol/L group, the fluorescence amplification curves demonstrated stronger signal intensity and earlier inflection points at higher concentrations. In summary, the 0.2/0.3 μmol/L concentration group demonstrated the best overall performance, with significantly reduced Ct values for HP-like PRRSV, stable amplification for NADC30-like PRRSV and NADC34-like PRRSV targets, and a balanced fluorescence signal. These findings indicate that this concentration provides optimal synergy between multi-target compatibility, detection sensitivity, and amplification efficiency (Figure 3).

### 3.4. Standard Curve Construction

**Figure 4:**
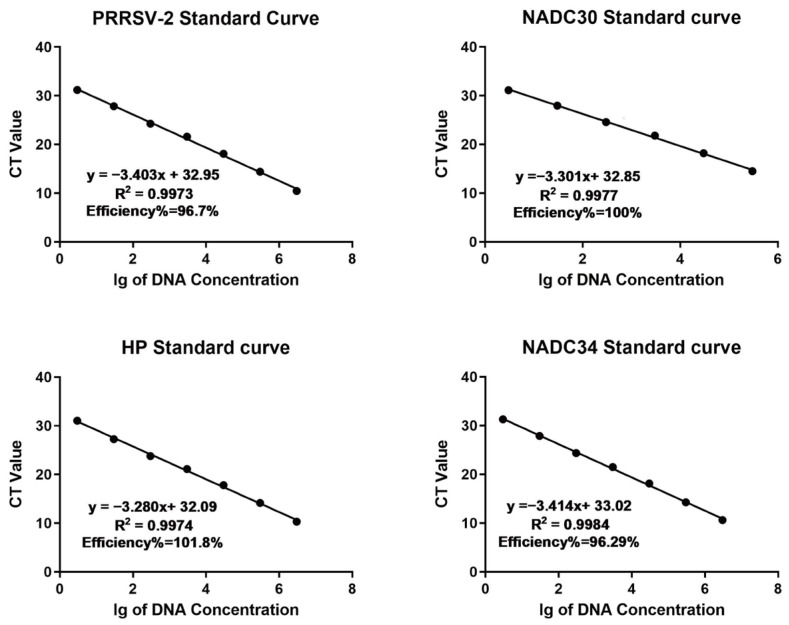
Standard curves were established for the four targets.

The established quadruplex RT-qPCR assay showed robust amplification dynamics across a standard plasmid concentration range of 3 to 3 × 10^6^ copies/μL for PRRSV-POS. The linear equations, correlation coefficients(R^2^), and amplification efficiencies of the standard curves were as follows: PRRSV-2: y = −3.403x + 32.95, R^2^ = 0.9973, E = 96.70%; NADC30-like PRRSV: y = −3.301x + 32.85, R^2^ = 0.9977, E = 100%; HP-like PRRSV: y = −3.280x + 32.09, R^2^ = 0.9974, E = 101.80%; NADC34-like PRRSV: y = −3.414x + 33.02, R^2^ = 0.9984, E = 96.29%. The results demonstrate that the slopes of the standard curves for different targets ranged from −3.414 to −3.280, with R^2^ all greater than 0.99, indicating a good linear relationship. The amplification efficiencies ranged from 96.29% to 101.80%, all meeting the qPCR methodological validation standards (Figure 4).

### 3.5. Sensitivity Test

**Figure 5:**
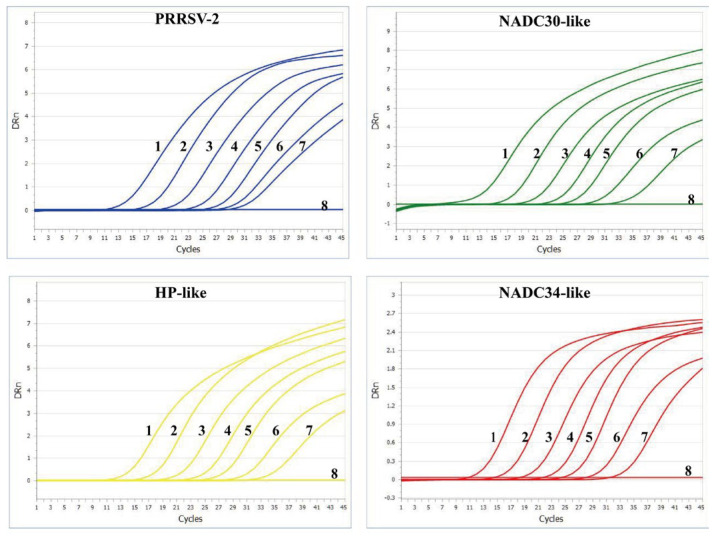
Sensitivity assay. PRRSV positive plasmid 1−7: 3–3 × 10^6^ copies/μL; 8: Negative control.

To evaluate the sensitivity of the RT-qPCR method established in this study, standard plasmids at concentrations ranging from 3 to 3 × 10^6^ copies/μL were tested. The quadruplex RT-qPCR assay demonstrated high sensitivity, with a limit of detection (LOD) of 3 copies/μL for all targets (Figure 5).

### 3.6. Specificity Test

**Figure 6:**
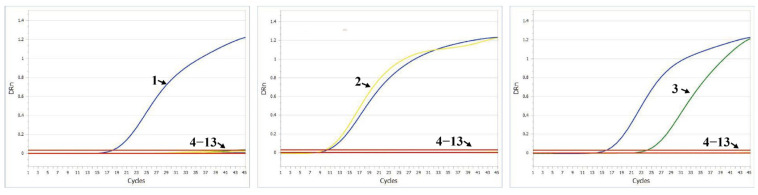
Specificity test of the PRRSV quadruplex RT-qPCR detection system against nine non-target porcine viruses. 1: C-PRRSV; 2: HP-like PRRSV; 3: NADC30-like PRRSV nucleic acid; 4−13: PEDV, TGEV, PDCoV, PoRV A, PPV, PRV, CSFV, PCV, JEV nucleic acid, and Nuclease-free H_2_O.

To verify the specificity of the established quadruplex RT-qPCR method, common porcine pathogens, including PPV, PRV, CSFV, PCV, JEV, PEDV, TGEV, PDCoV, and PoRV A, were used as DNA or RNA templates for the specificity test. C-PRRSV, NADC30-like PRRSV, and HP-like PRRSV nucleic acids were set as positive controls, and nuclease-free H_2_O was used as the negative control. The results showed that all positive control samples produced specific amplification curves, while the nine non-target pathogens and the negative control samples showed no cross-reaction signals (Figure 6). These results indicate that the method demonstrates high specificity for detecting various PRRSV-2 strains, with no cross-reactivity observed with other common porcine viruses.

### 3.7. Repeatability Test

To assess the stability and repeatability of the established quadruplex RT-qPCR method, standard plasmids at concentrations of 3 × 10^5^, 3 × 10^4^, and 3 × 10^3^ copies/μL were used as templates. The repeatability of the method was evaluated under both intra- and inter-batch conditions. The experimental data showed that the coefficient of variation (CV) intra-batches ranged from 0.03% to 1.48% (Table S5), while the inter-batches CV ranged from 0.15% to 1.31% (Table S6). These results indicate that the method demonstrates high stability and good repeatability under both different batches and the same batch conditions.

### 3.8. Concordance Validation Study

To evaluate the performance and accuracy of the established quadruplex RT-qPCR assay, a total of 389 clinical samples were tested in parallel using both the assay developed in this study and the national standard reference method for PRRSV detection. Among these samples, 37 were confirmed positive and 352 negative by both methods, demonstrating consistent diagnostic results. To further validate the lineage-typing accuracy of the assay, 26 of the RT-qPCR-positive samples were selected for sequencing of the NSP2 gene region, and the results fully matched the subtyping classifications obtained by RT-qPCR, including 11 NADC30-like, two NADC34-like, six HP-like, and seven Classical PRRSV strains (Table S7). This concordance supports the reliability of the assay for accurate genotyping across multiple PRRSV sublineages.

#### 3.8.1. Clinical Sample Testing from Farm A in Guangxi Zhuang Autonomous Region, China

**Figure 7:**
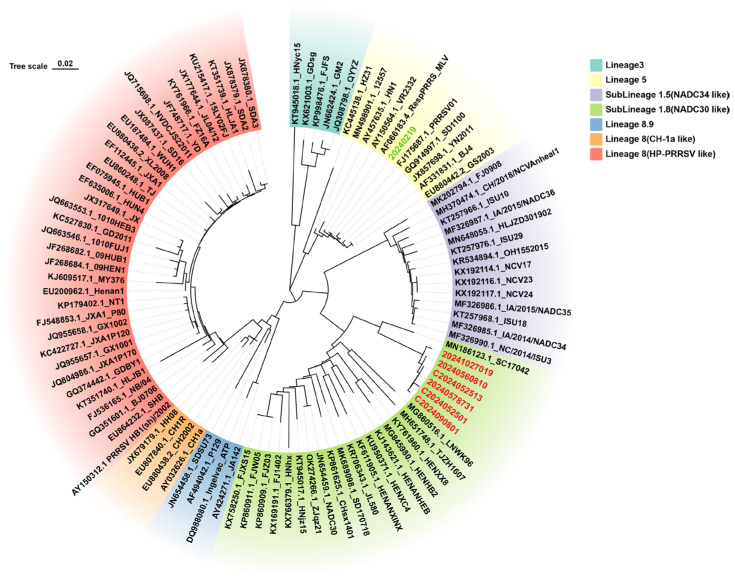
A phylogenetic tree was constructed using the Neighbor-Joining (NJ) method based on the ORF5 gene sequences of PRRSV-positive samples from Farm A, along with representative reference strains covering major PRRSV lineages, including classical PRRSV (C-PRRSV), HP-like PRRSV, NADC30-like PRRSV, and NADC34-like PRRSV. Farm A-derived lineage 1.8 NADC30-like strains are labeled in red font. Farm A-derived lineage 5 VR2332-like strains are labeled in green font.

**Figure 8:**
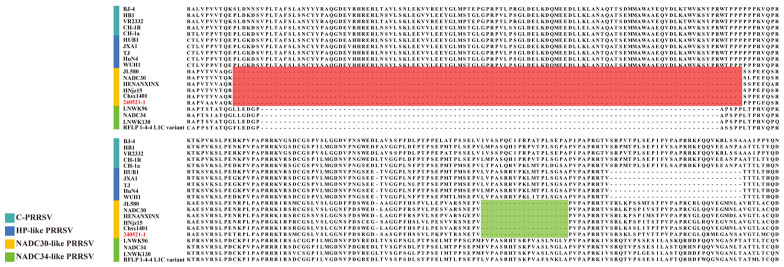
Amino acid sequence alignment of the NSP2 protein among PRRSV strains, including NADC30-like isolates from Farm A. The alignment compares the NSP2 amino acid sequences of PRRSV-positive samples from Farm A (indicated in red font) with representative strains from different PRRSV lineages. Strain lineages are indicated by colored bars in the left margin: cyan for C-PRRSV, blue for HP-like PRRSV, orange for NADC30-like PRRSV, and green for NADC34-like PRRSV. A discontinuous 131-amino acid deletion, characteristic of NADC30-like PRRSV, is evident in Farm A isolates, comprising a continuous deletion from amino acid positions 323 to 433, highlighted with a red box, and a second deletion from positions 504 to 522, marked with a green box.

To investigate whether field strains of PRRSV were circulating in a vaccinated farm, the method established in this study was applied for continuous surveillance of PRRSV prevalence at Farm A, a sow breeding farm in Guangxi Zhuang Autonomous Region, China. This farm uses a commercially available attenuated vaccine derived from a classical PRRSV strain for routine immunization. A total of 372 samples were tested, including testicular fluid, serum, abortion tissue, nasal swabs, and throat swabs. The PRRSV-2 detection rate was 21.50%, and the NADC30-like PRRSV detection rate was 13.44% (Table 2). Selected NADC30-like PRRSV-positive samples were subjected to ORF5 gene sequencing and phylogenetic tree construction. NSP2 gene sequencing was compared with the reference strain sequences, and the phylogenetic tree analysis revealed that the PRRSV strains at Farm A clustered with PRRSV-2 lineage 1 NADC30-like PRRSV strains (Figure 7). Further amino acid alignment of the NSP2 protein showed that all strains had a characteristic 131-amino-acid deletion at positions aa323–aa433, aa483, and aa504–aa522 (Figure 8). This molecular feature is consistent with the NADC30-like PRRSV strain. The results confirm the presence of NADC30-like PRRSV circulation in the vaccinated farm is consistent with the RT-qPCR detection results and indicate that the detection assay established in this study can be used for identifying and monitoring both vaccine and circulating strains on the farm. Detailed RT-qPCR results for the positive field samples are presented in Supplementary Materials Table S8.

#### 3.8.2. Clinical Sample Testing from Farm B in Guangxi Zhuang Autonomous Region, China

**Figure 9:**
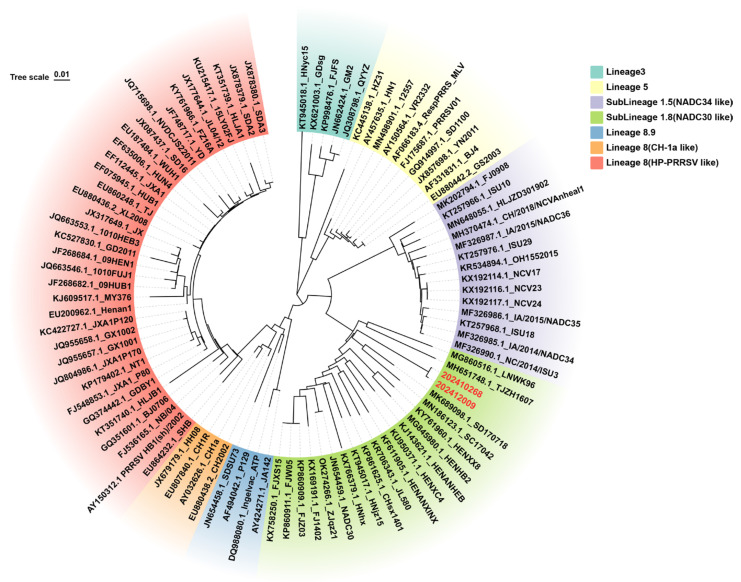
A phylogenetic tree was constructed using the Neighbor-Joining (NJ) method based on the ORF5 gene sequences of PRRSV-positive samples from Farm B, along with representative reference strains covering major PRRSV lineages, including classical PRRSV (C-PRRSV), HP-like PRRSV, NADC30-like PRRSV, and NADC34-like PRRSV. Farm B-derived NADC30-like strains are labeled in red font.

To investigate whether field strains of PRRSV were circulating in a vaccinated farm, the quadruplex RT-qPCR method was applied for continuous monitoring of the PRRSV prevalence at Farm B, a sow breeding farm in Guangxi Zhuang Autonomous Region, China. This farm has been using a commercially available attenuated vaccine derived from a classical PRRSV strain for immunization. A total of 566 samples were tested, including testicular fluid, serum, abortion tissue, nasal swabs, and throat swabs. The PRRSV-2 detection rate was 21.90%, while the NADC30-like PRRSV detection rate was 3.53% (Table 3). When classified by sample type, the results indicated that testicular fluid from neonatal piglets, abortion tissue, and nasal and throat swabs collected during routine monitoring all tested positive for C-PRRSV, indicating that the classical vaccine strain was the predominant virus circulating on the farm. Among 352 serum samples, 20 tested positive for NADC30-like PRRSV (Table 3). Some of the NADC30-like PRRSV-positive samples were selected for ORF5 gene sequencing and phylogenetic tree analysis, which showed that the PRRSV strains at Farm A clustered with PRRSV-2 lineage 1 NADC30-like PRRSV strains (Figure 9). This confirmed the presence of NADC30-like PRRSV strain circulation on the farm, which is in agreement with the detection results and demonstrates that the quadruplex RT-qPCR assay is effective for identifying and monitoring both vaccine-derived and field strains. Detailed RT-qPCR results for the positive field samples are presented in Supplementary Materials Table S9.

## 4. Discussion

PRRS is a highly contagious and economically devastating disease affecting the global swine industry [32]. In China, PRRSV shows significant genetic diversity, including both PRRSV-1 and PRRSV-2 genotypes, along with multiple co-circulating lineages. These include sublineage 1.8 (NADC30-like PRRSV), sublineage 1.5 (NADC34-like PRRSV), Lineage 3 (QYYZ-like PRRSV), Lineage 5 (VR2332-like PRRSV), and Lineage 8 (HP-PRRSV-like PRRSV), which frequently undergo recombination and rapid variation [33,34]. This genetic complexity presents considerable challenges for distinguishing PRRSV strains and developing reliable diagnostic and control strategies.

RT-qPCR addresses common limitations of conventional PCR by enabling closed-tube fluorescent signal monitoring. It offers high sensitivity for low viral load detection, reduced contamination risk, and quantitative viral load measurement through standard curve analysis. These advantages have led to the widespread adoption of RT-qPCR for PRRSV detection. To enhance discriminatory capacity, several multiplex assays have been developed. For instance, in 2019, Chen et al. established a TaqMan-MGB-based quadruplex RT-qPCR assay that simultaneously detects PRRSV-1, CA-PRRSV, HP-like PRRSV, and NADC30-like PRRSV with high specificity and a detection limit of 10^2^ copies/μL [35]. More recently, Ruan et al. (2023) developed an optimized quadruple RT-qPCR system that detects these strains with a sensitivity of 12 copies/μL [23]. However, these methods fail to further differentiate between NADC30-like PRRSV and NADC34-like PRRSV variants. Tu et al. (2023) addressed this gap by constructing a uniplex RT-qPCR targeting the ORF5 region of NADC34-like PRRSV, reaching a detection limit of 10¹ copies/μL with high specificity but lacking the multiplex capability needed to simultaneously detect other PRRSV-2 lineages [36]. In this study, a quadruplex RT-qPCR method was developed based on the molecular characteristics of strain-specific deletion patterns within the NSP2 gene of different PRRSV-2 strains. This assay enables the simultaneous detection of PRRSV-2 and differentiation among NADC30-like PRRSV, NADC34-like PRRSV, and HP-like PRRSV strains. The method showed high sensitivity (3 copies/μL), strong specificity without cross-reactivity to common swine pathogens, and good repeatability (CV < 1.5%). In field applications, the assay was successfully implemented in commercial swine farms immunized with an attenuated vaccine. The classical PRRSV vaccine strain was detected across multiple sample types, indicating its continued circulation. ORF5 sequencing confirmed that NADC30-like positive samples clustered within lineage 1, validating the assay’s accuracy. These results demonstrate the effectiveness of the assay for identifying and monitoring both vaccine-derived and circulating PRRSV strains.

In this study, both farms used classical PRRSV vaccines derived from lineage 5, which may explain the similar PRRSV-2 positivity rates observed at Farm A (21.50%) and Farm B (21.90%). However, the detection rate of NADC30-like PRRSV strains was notably higher at Farm A (13.44%) compared to Farm B (3.53%), primarily due to more positive samples identified in testicular fluids from piglets at Farm A. NADC34-like PRRSV, HP-like PRRSV, and PRRSV-1 strains were not detected in either farm. These results are consistent with Fang et al. (2022), who reported that PRRSV-2 lineage 1 (NADC30-like PRRSV) was the second most prevalent genotype (13.78%) in South China from 2017 to 2021 [37]. These findings highlight the regional predominance of NADC30-like PRRSV strains. The absence of HP-like PRRSV and NADC34-like PRRSV, PRRSV-1 strain detections in our field samples may reflect their lower prevalence in the tested farms rather than a failure of the assay. Nevertheless, further validation using samples from different geographic regions and broader epidemiological contexts is necessary to confirm the assay’s effectiveness for these lineages.

The NSP2 gene of PRRSV is one of the most genetically variable regions in the PRRSV genome, frequently undergoing deletions, insertions, and recombination events. Since 2014, sublineage 1.8 (NADC30-like PRRSV) has gradually emerged as the predominant lineage involved in PRRSV recombination, accounting for approximately 63.7% of all recombinant strains [38]. Several studies have identified recombination break-points and high sequence diversity within the NSP2 coding region [39,40], which may lead to inconsistencies between ORF5- and NSP2-based lineage classification and compromise the accuracy of genotyping and clinical diagnostics. Additionally, the primers and probes that target a specific deletion characteristic are located within the highly variable region of the PRRSV NSP2 gene. Consequently, novel recombination or mutation events within this region may compromise the recognition efficiency of the primers and probes. In addition, the current method is limited to targeting only the NSP2 gene fragment and does not include information from other genomic regions. Despite these challenges, the deletion patterns in the NSP2 gene remain valuable for distinguishing between classical PRRSV vaccine strains (either C-PRRSV or HP-like PRRSV strains) and the currently prevalent NADC30-like PRRSV and NADC34-like PRRSV variants. These markers continue to support molecular epidemiology and optimize immunization strategies. To enhance the long-term applicability of the assay, future work will involve periodic sequence monitoring of circulating PRRSV strains to support timely updates of the primer and probe design and ensure continued assay reliability.

To effectively manage the persistent threat of PRRSV-2, a comprehensive strategy that combines accurate diagnostics with standard on-farm control measures is essential. Biosecurity remains the cornerstone of prevention and includes practices such as isolating replacement gilts, sanitizing vehicles, and using air filtration systems [41]. Herd management strategies based on McREBEL principles and all-in/all-out production systems have proven effective in reducing virus transmission within and between litters [42]. Early surveillance is equally important and involves sampling oral and processing fluids, performing viral genotyping, and applying GIS-based modeling to predict outbreak risks [43]. Although vaccination remains a primary tool for PRRSV prevention, its effectiveness may be constrained by genetic divergence between field and vaccine strains. Aligning genotyping data with vaccine selection enhances the precision and effectiveness of immunization programs. The integration of accurate detection methods, strict biosecurity, optimized herd management, and continued epidemiological surveillance offers a solid foundation for a more sustainable and adaptive PRRSV control system.

## 5. Conclusions

This study developed a quadruplex RT-qPCR method targeting NSP2 gene deletions to simultaneously detect PRRSV-2 and differentiate NADC30-like PRRSV, HP-like PRRSV, and NADC34-like PRRSV strains. The assay demonstrated high sensitivity, strong specificity with no cross-reactivity to common swine pathogens, and good repeatability. Results from laboratory validation were consistent with sequencing, confirming the diagnostic accuracy of the method. Field application in two Guangxi farms supported its effectiveness in detecting circulating and vaccine-derived NADC30-like PRRSV strains. While the assay demonstrated the capability to detect and correctly subtype NADC30-like PRRSV, HP-like PRRSV, and NADC34-like PRRSV strains in clinical samples, field validation for HP-like and NADC34-like PRRSV remains limited. Further studies using broader and more geographically diverse sample sets are required to fully confirm the assay’s applicability across the predominant genetic sublineages of circulating PRRSV-2.

## Figures and Tables

**Table 1:** Primer/probe sets designed in this study.

Target	Primer/Probe Sets	Sequences (5′-3′)	Locations (nt)	Amplicon Size
PRRSV 2 (ORF7/3′UTR)	PRRSV-2-F	GCACTGATTGACAYTGTGCC	15,317–15,336	85 bp
	PRRSV-2-R	CGCATGGTTCTCGCCAAT	15,384–15,401	
	PRRSV-2-P	AGTCACCTATTCAATTAGGGCGACCG	15,341–15,366	
NADC30-like PRRSV (NSP2)	NADC30-like-F	AGCGTRYTGGAYACCTCCTTTG	2178–2199	212 bp
	NADC30-like-R	CTGATYTTYCTGCGYGGRG	2371–2389	
	NADC30-like-P	TTCTCTGGRAGGCTCCTGACAGACYC	2331–2356	
HP-like PRRSV (NSP2)	HP-PRRSV-F	TGGGTCGGCRCCAKTTCCT	2892–2910	101 bp
	HP-PRRSV-R	YATATTCCGTYTGTGAGGACRC	2916–2941	
	HP-PRRSV-P	TCAGCGTTGTTGTYACAGTYCTRCGC	2971–2992	
NADC34-like PRRSV (NSP2)	NADC34-like-F	AGTYCACCTAACYGAGTTRCC	2231–2251	169 bp
	1-4-4-F	AGTTCACYYAACCAAGCTGCC	2189–2209	
	NADC34-like-R	AGGGRYGGGCTTGCAATCT	2381–2399	
	NADC34-like-P	CGGCCYCATCGCCRCCTCT	2314–2332	

**Table 2:** RT-qPCR results and positive rates of clinical samples from Farm A.

Target	Testicular Fluid	Serum	Tissue	Oral and Nasal Swabs	Total	Positive Rate (%)
PRRSV-2	28	47	4	1	80	21.50
NADC30-like PRRSV	17	29	4	0	50	13.44
HP-like PRRSV	0	0	0	0	0	0
NADC34-like PRRSV	0	0	0	0	0	0
Total	59	240	45	28	372	-

**Table 3:** RT-qPCR results and positive rates of clinical samples from Farm B.

Target	Testicular Fluid	Serum	Tissue	Oral and Nasal Swabs	Total	Positive Rate(%)
PRRSV-2	26	68	21	9	124	21.90
NADC30-like PRRSV	0	20	0	0	20	3.53
HP-like PRRSV	0	0	0	0	0	0
NADC34-like PRRSV	0	0	0	0	0	0
Total	109	352	45	60	566	-

## Data Availability

The original contributions presented in this study are included in the article. Further inquiries can be directed to the corresponding author.

## Supplementary Materials

The following supporting information can be downloaded at: https://www.mdpi.com/article/10.3390/v17060853/s1, Table S1: Standard Plasmid Sequences; Table S2: RT-qPCR reaction system; Table S3: RT-qPCR reaction program; Table S4: Primer Targeting ORF5 and NSP2 Genes; Table S5: Intra-batch reproducibility assessment; Table S6: Inter-batch reproducibility assessment; Table S7: Comparison of the consistency between the quadruplex RT-qPCR method and sequencing results; Table S8: Ct values of PRRSV-positive clinical samples detected by the quadruplex RT-qPCR assay in breeding Farm A, Guangxi Zhuang Autonomous Region; Table S9: Ct values of PRRSV-positive clinical samples detected by the quadruplex RT-qPCR assay in breeding Farm B, Guangxi Zhuang Autonomous Region.

## Abbreviations

The following abbreviations are used in this manuscript:

PRRSV	Porcine reproductive and respiratory syndrome virus
C-PRRSV	Classic strains—Porcine reproductive and respiratory syndrome virus
HP-PRRSV	Highly pathogenic strains—Porcine reproductive and respiratory syndrome virus
PEDV	Porcine epidemic diarrhea virus
PoRV A	Group A porcine rotavirus
PDCoV	Porcine deltacoronavirus
PRV	Pseudorabies virus
CSFV	Classical swine fever virus
PPV	Porcine parvovirus
LOD	The limit of detection
CV	Coefficient of variation
aa	Amino acids

## References

[1] Collins J.E., Benfield D.A., Christianson W.T., Harris L., Hennings J.C., Shaw D.P., Goyal S.M., McCullough S., Morrison R.B., Joo H.S. (1992). Isolation of Swine Infertility and Respiratory Syndrome Virus (Isolate ATCC VR-2332) in North America and Experimental Reproduction of the Disease in Gnotobiotic Pigs. J. Vet. Diagn. Investig..

[2] de Almeida M.N., Corzo C.A., Zimmerman J.J., Linhares D.C.L. (2021). Longitudinal Piglet Sampling in Commercial Sow Farms Highlights the Challenge of PRRSV Detection. Porc. Health Manag..

[3] Karniychuk U.U., Nauwynck H.J. (2009). Quantitative Changes of Sialoadhesin and CD163 Positive Macrophages in the Implantation Sites and Organs of Porcine Embryos/Fetuses During Gestation. Placenta.

[4] Huang B., Li F., You D., Deng L., Xu T., Lai S., Ai Y., Huang J., Zhou Y., Ge L. (2024). Porcine Reproductive and Respiratory Syndrome Virus Infects the Reproductive System of Male Piglets and Impairs Development of the Blood–Testis Barrier. Virulence.

[5] Cai H., Zhang H., Cheng H., Liu M., Wen S., Ren J. (2023). Progress in PRRSV Infection and Adaptive Immune Response Mechanisms. Viruses.

[6] Zhang J., Wang P., Xie C., Ha Z., Shi N., Zhang H., Li Z., Han J., Xie Y., Qiu X. (2022). Synergistic Pathogenicity by Coinfection and Sequential Infection with NADC30-like PRRSV and PCV2 in Post-Weaned Pigs. Viruses.

[7] Holtkamp D.J., Kliebenstein J.B., Zimmerman J.J., Neumann E., Rotto H., Yoder T.K., Wang C., Yeske P., Mowrer C.L., Haley C. (2012). Economic Impact of Porcine Reproductive and Respiratory Syndrome Virus on U.S. Pork Producers.

[8] Zhang Z., Li Z., Li H., Yang S., Ren F., Bian T., Sun L., Zhou B., Zhou L., Qu X. (2022). The Economic Impact of Porcine Reproductive and Respiratory Syndrome Outbreak in Four Chinese Farms: Based on Cost and Revenue Analysis. Front. Vet. Sci..

[9] Tian K., Yu X., Zhao T., Feng Y., Cao Z., Wang C., Hu Y., Chen X., Hu D., Tian X. (2007). Emergence of Fatal PRRSV Variants: Unparalleled Outbreaks of Atypical PRRS in China and Molecular Dissection of the Unique Hallmark. PLoS ONE.

[10] Brockmeier S.L., Loving C.L., Vorwald A.C., Kehrli M.E., Baker R.B., Nicholson T.L., Lager K.M., Miller L.C., Faaberg K.S. (2012). Genomic Sequence and Virulence Comparison of Four Type 2 Porcine Reproductive and Respiratory Syndrome Virus Strains. Virus Res..

[11] Zhou L., Wang Z., Ding Y., Ge X., Guo X., Yang H. (2015). NADC30-like Strain of Porcine Reproductive and Respiratory Syndrome Virus, China. Emerg. Infect. Dis. J. CDC.

[12] Zhou L., Yang B., Xu L., Jin H., Ge X., Guo X., Han J., Yang H. (2017). Efficacy Evaluation of Three Modified-Live Virus Vaccines against a Strain of Porcine Reproductive and Respiratory Syndrome Virus NADC30-Like. Vet. Microbiol..

[13] Van Geelen A.G.M., Anderson T.K., Lager K.M., Das P.B., Otis N.J., Montiel N.A., Miller L.C., Kulshreshtha V., Buckley A.C., Brockmeier S.L. (2018). Porcine Reproductive and Respiratory Disease Virus: Evolution and Recombination Yields Distinct ORF5 RFLP 1-7-4 Viruses with Individual Pathogenicity. Virology.

[14] Zhang H.-L., Zhang W.-L., Xiang L.-R., Leng C.-L., Tian Z.-J., Tang Y.-D., Cai X.-H. (2018). Emergence of Novel Porcine Reproductive and Respiratory Syndrome Viruses (ORF5 RFLP 1-7-4 Viruses) in China. Vet. Microbiol..

[15] Xie C.-Z., Ha Z., Zhang H., Zhang Y., Xie Y.-B., Zhang H., Nan F.-L., Wang Z., Zhang P., Xu W. (2020). Pathogenicity of Porcine Reproductive and Respiratory Syndrome Virus (ORF5 RFLP 1-7-4 Viruses) in China. Transbound. Emerg. Dis..

[16] Gong B., Xu H., Sun Q., Li C., Xiang L., Zhao J., Li W., Guo Z., Li J., Wang Q. (2024). Dissecting Genetic Diversity and Evolutionary Trends of Chinese PRRSV-1 Based on Whole-Genome Analysis. Transbound. Emerg. Dis..

[17] Ming S., Yongying M., Bohua L., Huiying L., Xiaoyu D., Qiaorong L., Mingming Q., Xi C., Xinyan Y., Xizhao C. (2017). Pathogenic Characterization of European Genotype Porcine Reproductive and Respiratory Syndrome Virus Recently Isolated in Mainland China. Open Virol. J..

[18] Hsueh F.-C., Kuo K.-L., Hsu F.-Y., Wang S.-Y., Chiu H.-J., Wu M.-T., Lin C.-F., Huang Y.-H., Chiou M.-T., Lin C.-N. (2023). Molecular Characteristics and Pathogenicity of Porcine Reproductive and Respiratory Syndrome Virus (PRRSV) 1 in Taiwan during 2019–2020. Life.

[19] Wang X., Bai X., Wang Y., Wang L., Wei L., Tan F., Zhou Z., Tian K. (2023). Pathogenicity Characterization of PRRSV-1 181187-2 Isolated in China. Microb. Pathog..

[20] Xu H., Gong B., Sun Q., Li C., Zhao J., Xiang L., Li W., Guo Z., Tang Y., Leng C. (2023). Genomic Characterization and Pathogenicity of BJEU06-1-Like PRRSV-1 ZD-1 Isolated in China. Transbound. Emerg. Dis..

[21] Yang S., Cui M., Li C., Qiu M., Zhu X., Lin Y., Meng Y., Qiu Y., Qi W., Lin H. (2025). Isolation and Genomic Characterization of a Novel Porcine Reproductive and Respiratory Syndrome Virus 1 from Severely Diseased Piglets in China in 2024. Vet. Sci..

[22] Li Y., Ji G., Xu X., Wang J., Li Y., Tan F., Li X. (2017). Development and Application of an RT-PCR to Differentiate the Prevalent NA-PRRSV Strains in China. Open Virol. J..

[23] Ruan S., Ren W., Yu B., Yu X., Wu H., Li W., Jiang Y., He Q. (2023). Development and Implementation of a Quadruple RT-qPCR Method for the Identification of Porcine Reproductive and Respiratory Syndrome Virus Strains. Viruses.

[24] Park J.-Y., Park S., Park Y.-R., Kang D.-Y., Kim E.-M., Jeon H.-S., Kim J.-J., Kim W.-I., Lee K.-T., Kim S.-H. (2016). Reverse-Transcription Loop-Mediated Isothermal Amplification (RT-LAMP) Assay for the Visual Detection of European and North American Porcine Reproductive and Respiratory Syndrome Viruses. J. Virol. Methods.

[25] Yang Y., Qin X., Sun Y., Chen T., Zhang Z. (2016). Rapid Detection of Highly Pathogenic Porcine Reproductive and Respiratory Syndrome Virus by a Fluorescent Probe-Based Isothermal Recombinase Polymerase Amplification Assay. Virus Genes.

[26] Long F., Chen Y., Shi K., Yin Y., Feng S., Si H. (2023). Development of a Multiplex Crystal Digital RT-PCR for Differential Detection of Classical, Highly Pathogenic, and NADC30-like Porcine Reproductive and Respiratory Syndrome Virus. Animals.

[27] Liu S., Tao D., Liao Y., Yang Y., Sun S., Zhao Y., Yang P., Tang Y., Chen B., Liu Y. (2021). Highly Sensitive CRISPR/Cas12a-Based Fluorescence Detection of Porcine Reproductive and Respiratory Syndrome Virus. ACS Synth. Biol..

[28] Wang X., Wang G., Wang N., Liu J., Cai Y., Ren M., Li Z. (2019). A Simple and Efficient Method for the Generation of a Porcine Alveolar Macrophage Cell Line for High-Efficiency Porcine Reproductive and Respiratory Syndrome Virus 2 Infection. J. Virol. Methods.

[29] Singh C., Roy-Chowdhuri S., Luthra R., Singh R.R., Patel K.P. (2016). Quantitative Real-Time PCR: Recent Advances. Clinical Applications of PCR.

[30] Xie J., Chen Y., Cai G., Cai R., Hu Z., Wang H. (2023). Tree Visualization By One Table (tvBOT): A Web Application for Visualizing, Modifying and Annotating Phylogenetic Trees. Nucleic Acids Res..

[31] (2023). Diagnostic Techniques for Porcine Reproductive and Respiratory Syndrome.

[32] Hu R., Zhang T., Lai R., Ding Z., Zhuang Y., Liu H., Cao H., Gao X., Luo J., Chen Z. (2023). PRRSV Elimination in a Farrow-to-Finish Pig Herd Using Herd Closure and Rollover Approach. Viruses.

[33] Yim-im W., Anderson T.K., Paploski I.A.D., VanderWaal K., Gauger P., Krueger K., Shi M., Main R., Zhang J. (2023). Refining PRRSV-2 Genetic Classification Based on Global ORF5 Sequences and Investigation of Their Geographic Distributions and Temporal Changes. Microbiol. Spectr..

[34] Zhao Y.-Y., Ma X., Chen X.-M., Song Y.-P., Zheng L.-L., Ma S.-J., Chen H.-Y. (2024). Molecular Detection and Genetic Characteristics of Porcine Reproductive and Respiratory Syndrome Virus in Central China. Microb. Pathog..

[35] Chen N., Ye M., Xiao Y., Li S., Huang Y., Li X., Tian K., Zhu J. (2019). Development of Universal and Quadruplex Real-time RT-PCR Assays for Simultaneous Detection and Differentiation of Porcine Reproductive and Respiratory Syndrome Viruses. Transbound Emerg. Dis..

[36] Tu T., Pang M., Jiang D., Zhou Y., Wu X., Yao X., Luo Y., Yang Z., Ren M., Lu A. (2023). Development of a Real-Time TaqMan RT-PCR Assay for the Detection of NADC34-like Porcine Reproductive and Respiratory Syndrome Virus. Vet. Sci..

[37] Fang K., Liu S., Li X., Chen H., Qian P. (2022). Epidemiological and Genetic Characteristics of Porcine Reproductive and Respiratory Syndrome Virus in South China Between 2017 and 2021. Front. Vet. Sci..

[38] Zhang R., Li H., Xie H., Hou X., Zhou L., Cao A., Zeshan B., Zhou Y., Wang X. (2024). Comparing the Molecular Evolution and Recombination Patterns of Predominant PRRSV-2 Lineages Co-Circulating in China. Front. Microbiol..

[39] Zhang H., Luo Q., Zheng Y., Sha H., Li G., Kong W., Huang L., Zhao M. (2023). Genetic Variability and Recombination of the NSP2 Gene of PRRSV-2 Strains in China from 1996 to 2021. Vet. Sci..

[40] Liu B., Luo L., Shi Z., Ju H., Yu L., Li G., Cui J. (2023). Research Progress of Porcine Reproductive and Respiratory Syndrome Virus NSP2 Protein. Viruses.

[41] Otake S., Yoshida M., Dee S. (2024). A Review of Swine Breeding Herd Biosecurity in the United States to Prevent Virus Entry Using Porcine Reproductive and Respiratory Syndrome Virus as a Model Pathogen. Animals.

[42] Chen P., Wu H., Wang X. (2024). Effects of Herd Closure and Medication Programs on the Infection of NADC30-like PRRSV in Pig Farms. J. Vet. Sci..

[43] Magalhães E.S., Zimmerman J.J., Holtkamp D.J., Classen D.M., Groth D.D., Glowzenski L., Philips R., Silva G.S., Linhares D.C.L. (2021). Next Generation of Voluntary PRRS Virus Regional Control Programs. Front. Vet. Sci..

