# Supplementary material for: Development of a Quadruplex RT-qPCR Assay for Rapid Detection and Differentiation of PRRSV-2 and Its Predominant Genetic Sublineages in China
Source: Viruses. 2025 Jun 16;17(6):853. doi: 10.3390/v17060853 (PMC12197470; doi:10.3390/v17060853)
Supplement: Supplementary file 1 [file viruses-17-00853-s001.zip › viruses-3622911-supplementary.pdf]

Supplementary Materials

Table S1. Standard Plasmid Sequences

Standard plasmids	Sequence
PRRSV-POS	GCACTGATTGATATTGTGCCTTAGTCACCTATTCAATTAGGGC GACCGTTATTGGCGAGAACCATGCGTTAGCGTATTGGACACC TCCTTTGTTGAGTCTGTCAGGAGCCTTCCAGAGAATTCCCCA CGCAGGAAGATCAGTTTGGGTCGGCACCAGTTCCTTTGCGCA GAACTGTGACAACAACGCTGATTGCGTCCTCACAGACGGAA TATGTTAGTTCACTCAACCAAGCTGCCTTAGTTCACCTAACC GAGTTGCCTTCGGCCCCATCGCCACCTCTTTAGATTGCAAGC CCGTCCCTTTCAGATGCAGATTGTGTTGCCTTTATACATTCTG GCCCCTGCCCATCACGTTTGAAAGTGCTGCAGGTCTCCA

Table S2. RT-qPCR reaction system

Component	Volume（μL）
5×One Step U+ MIX	6.0
One Step U+ Enzyme MIX	1.5
PRRSV-2-F（10μM）	0.6
PRRSV-2-R（10μM）	0.6
PRRSV-2-P（10μM）	0.9
NADC30-like -F（10μM）	0.6
NADC30-like -R（10μM）	0.6
NADC30-like -P（10μM）	0.9
HP-PRRSV-F（10μM）	0.6
HP-PRRSV-R（10μM）	0.6
HP-PRRSV-P（10μM）	0.9
NADC34-like -F（10μM）	0.6
1-4-4-F（10μM）	0.6
NADC34-like-R（10μM）	0.6
NADC34-like-P（10μM）	0.9
Template	5.0
Nuclease-free H_2O	8.5
Total	30

Table S3. RT-qPCR reaction program

Reaction Stage	Reaction Temperature	Response Time	Cycles
Reverse transcription	55℃	15min	1
Initial denaturation	95℃	30sec	1
Denaturation	95℃	10sec	45
Annealing/Extension	56/58/60℃	30sec	45

Table S4. Primer targeting ORF5 and NSP2 genes

Target	Sequences (5′-3′)
ORF5-F	GAGGTGGGCAACYGTTTTAG
ORF5-R	CAMGMGTAGCGCCAGGACA
NSP2-F	ATTGGRATGTTGTGYTYCCTGG
NSP2-R	GAGCTGARTAYTTTGGGCGYG

Table S5. Intra-batch reproducibility assessment

Target	Standard Plasmid（copies/μL）	Ct Value			$\bar{x}\pm S$	CV（%）
		1	2	3		
PRRSV-2	3×10^5	21.34	21.33	21.33	21.33±0.01	0.03
	3×10^4	25.3	25.45	25.55	25.43±0.13	0.49
	3×10^3	29.31	29.41	29.1	29.27±0.16	0.54
NADC30-Like	3×10^5	20.77	20.95	20.86	20.86±0.09	0.43
	3×10^4	25.18	25.12	25.02	25.11±0.08	0.32
	3×10^3	29.19	29.27	29.98	29.48±0.43	1.48
HP-like	3×10^5	20.79	20.82	20.78	20.80±0.02	0.1
	3×10^4	24.83	24.87	24.88	24.86±0.03	0.11
	3×10^3	29.07	29	29.03	29.03±0.04	0.12
NADC34-Like	3×10^5	20.12	20.05	20.11	20.09±0.04	0.19
	3×10^4	24.03	24.15	24.17	24.12±0.08	0.31
	3×10^3	28.49	28.5	28.54	28.51±0.03	0.09

Table S6. Inter-batch reproducibility assessment

Target	Standard Plasmid (copies/μL)	Ct Value					$\bar{x}$±S	CV (%)
		1	2	3	4	5		
PRRSV-2	3×10^5	21.33	21.36	21.28	21.36	21.35	21.34±0.03	0.15
	3×10^4	25.43	25.49	25.40	25.38	25.46	25.43±0.05	0.18
	3×10^3	29.27	29.72	29.48	29.48	29.57	29.50± 0.16	0.55
NADC30-like-PRRSV	3×10^5	20.86	20.73	20.70	20.81	20.89	20.80± 0.08	0.40
	3×10^4	25.02	25.11	25.03	24.98	25.06	25.04± 0.05	0.19
	3×10^3	28.74	29.48	29.49	29.04	29.67	29.28± 0.38	1.31
HP-like-PRRSV	3×10^5	20.80	20.84	20.74	20.89	20.96	20.85± 0.08	0.40
	3×10^4	24.91	24.69	24.85	24.59	24.70	24.75± 0.13	0.53
	3×10^3	28.85	29.03	28.77	28.68	29.13	28.89± 0.18	0.64
NADC34-like-PRRSV	3×10^5	20.09	20.04	20.11	20.16	20.19	20.12± 0.06	0.29
	3×10^4	24.12	24.10	24.07	24.06	24.01	24.07±0.04	0.18
	3×10^3	28.33	28.51	28.36	28.34	28.47	28.40± 0.08	0.29

Table S7. Comparison of the consistency between the quadruplex RT-qPCR method and sequencing results

Sample Number	PRRSV-2 Fam	NADC30-like Hex	HP-like Rox	NADC34-like Cy5	Sequenced results
1	21.00	26.44	N	N	NADC30-like
2	16.03	24.97	N	N	NADC30-like
3	12.96	23.37	N	N	NADC30-like
4	19.15	23.37	N	N	NADC30-like
5	25.75	30.91	N	N	NADC30-like
6	17.79	22.27	N	N	NADC30-like
7	23.07	31.28	N	N	NADC30-like
8	27.10	N	N	N	C-PRRSV
9	24.66	33.62	N	N	NADC30-like
10	19.51	N	N	N	C-PRRSV
11	26.90	N	N	N	C-PRRSV
12	24.96	N	N	N	C-PRRSV
13	20.08	28.10	N	N	NADC30-like
14	19.34	26.93	N	N	NADC30-like
15	19.63	26.91	N	N	NADC30-like
16	20.80	N	N	24.21	NADC34-like
17	20.92	N	N	24.26	NADC34-like
18	24.23	N	N	N	C-PRRSV
19	26.84	N	N	N	C-PRRSV

20	27.92	N	N	N	C-PRRSV
21	15.43	N	22.95	N	HP-like
22	10.23	N	10.09	N	HP-like
23	18.16	N	25.18	N	HP-like
24	26.07	N	24.23	N	HP-like
25	25.70	N	23.84	N	HP-like
26	14.72	N	26.43	N	HP-like

Table S8. Ct values of PRRSV-positive clinical samples detected by the quadruplex RT-qPCR assay in breeding Farm A, Guangxi Zhuang Autonomous Region

Number	Sample type	PRRSV-2	NADC30-like	HP-like	NADC34-like
1	Serum	39.32	27.16	N	N
2	Testicular Fluid	38.45	29.88	N	N
3	Testicular Fluid	39.46	28.11	N	N
4	Serum	27.95	N	N	N
5	Serum	27.01	24.98	N	N
6	Serum	33.34	29.57	N	N
7	Serum	31.29	27.95	N	N
8	Testicular Fluid	35.082	26.23	N	N
9	Testicular Fluid	30.324	25.27	N	N
10	Testicular Fluid	27.23	22.824	N	N
11	Testicular Fluid	20.57	31.96	N	N
12	Serum	23.02	N	N	N
13	Serum	19.78	24.6	N	N
14	Serum	25.03	N	N	N
15	Serum	24.37	N	N	N
16	Serum	16.473	21.582	N	N
17	Serum	17.879	22.902	N	N
18	Serum	24.32	N	N	N
19	Serum	24.43	N	N	N
20	Serum	17.79	22.27	N	N
21	Serum	25.75	30.91	N	N
22	Serum	19.15	23.37	N	N
23	Tissue	12.96	23.37	N	N
24	Testicular Fluid	39.86	26.96	N	N
25	Testicular Fluid	37.66	26.82	N	N
26	Testicular Fluid	21.55	N	N	N
27	Testicular Fluid	21.65	N	N	N
28	Serum	25.33	30.23	N	N
29	Serum	24.41	29.37	N	N
30	Serum	22.84	27.52	N	N
31	Serum	22.27	26.55	N	N
32	Serum	15.66	20.53	N	N

33	Serum	15.61	20.54	N	N
34	Serum	15.57	20.55	N	N
35	Testicular Fluid	25.223	37.129	N	N
36	Testicular Fluid	30.99	N	N	N
37	Testicular Fluid	26.39	39.59	N	N
38	Testicular Fluid	30.26	N	N	N
39	Testicular Fluid	19.85	25.31	N	N
40	Serum	25.93	31.34	N	N
41	Serum	27.82	33.38	N	N
42	Serum	20.074	25.543	N	N
43	Serum	30.918	N	N	N
44	Serum	31.207	N	N	N
45	Tissue	12.72	27.28	N	N
46	Serum	16.4	21.24	N	N
47	Oral and Nasal swabs	28	N	N	N
48	Testicular Fluid	22.83	33.97	N	N
49	Tissue	16.03	24.97	N	N
50	Testicular Fluid	27.1	N	N	N
51	Serum	34.09	N	N	N
52	Serum	38.88	N	N	N
53	Testicular Fluid	35.95	N	N	N
54	Testicular Fluid	33.8	N	N	N
55	Testicular Fluid	36.74	N	N	N
56	Testicular Fluid	36.81	N	N	N
57	Testicular Fluid	23.07	31.28	N	N
58	Testicular Fluid	30.97	N	N	N
59	Testicular Fluid	24.66	33.62	N	N
60	Serum	21	26.44	N	N
61	Testicular Fluid	20.41	26.06	N	N
62	Testicular Fluid	25.36	N	N	N
63	Testicular Fluid	23.65	31.99	N	N
64	Serum	16.22	20.83	N	N
65	Serum	24.17	29.07	N	N
66	Serum	36.21	N	N	N
67	Serum	36.55	N	N	N
68	Serum	36.65	N	N	N
69	Serum	34.63	N	N	N
70	Serum	33.74	N	N	N
71	Tissue	30.35	38.06	N	N
72	Serum	31.01	31.4	N	N
73	Serum	22.23	27.88	N	N
74	Serum	33.41	32.79	N	N
75	Testicular Fluid	23.71	28.51	N	N

76	Serum	35.4	N	N	N
77	Serum	32.6	N	N	N
78	Serum	20.47	25.12	N	N
79	Serum	17.43	22.06	N	N
80	Serum	28.63	N	N	N

Table S9. Ct values of PRRSV-positive clinical samples detected by the quadruplex RT-qPCR assay in breeding Farm B, Guangxi Zhuang Autonomous Region

Number	Sample type	PRRSV-2	NADC30-like	HP-like	NADC34-like
1	Serum	30.26	N	N	N
2	Serum	26.94	N	N	N
3	Serum	43.68	N	N	N
4	Serum	32.68	N	N	N
5	Serum	17.46	31.04	N	N
6	Serum	36.25	N	N	N
7	Serum	38.07	N	N	N
8	Serum	24.23	37.42	N	N
9	Serum	32.3	N	N	N
10	Serum	29.23	N	N	N
11	Testicular Fluid	28.26	N	N	N
12	Testicular Fluid	30.56	N	N	N
13	Testicular Fluid	29.76	N	N	N
14	Testicular Fluid	30.57	N	N	N
15	Serum	21.28	N	N	N
16	Serum	28.43	N	N	N
17	Serum	29.36	N	N	N
18	Serum	20.08	28.1	N	N
19	Serum	19.34	26.93	N	N
20	Serum	19.63	26.91	N	N
21	Serum	22.65	33.35	N	N
22	Serum	22.03	31.64	N	N
23	Testicular Fluid	24.23	N	N	N
24	Testicular Fluid	26.84	N	N	N
25	Testicular Fluid	27.92	N	N	N
26	Serum	39.05	N	N	N
27	Serum	32.01	N	N	N
28	Serum	36.11	N	N	N
29	Serum	34.59	N	N	N
30	Serum	35.46	N	N	N
31	Serum	30.621	N	N	N
32	Serum	32.645	N	N	N

33	Oral and Nasal swabs	34.12	N	N	N
34	Oral and Nasal swabs	37.2	N	N	N
35	Oral and Nasal swabs	34.34	N	N	N
36	Oral and Nasal swabs	36.42	N	N	N
37	Oral and Nasal swabs	36.3	N	N	N
38	Oral and Nasal swabs	30.26	N	N	N
39	Serum	34.22	N	N	N
40	Serum	32.08	N	N	N
41	Serum	33.6	N	N	N
42	Testicular Fluid	38.2	N	N	N
43	Testicular Fluid	28.2	N	N	N
44	Testicular Fluid	33.63	N	N	N
45	Tissue	35.02	N	N	N
46	Tissue	19.51	N	N	N
47	Testicular Fluid	26.9	N	N	N
48	Serum	38.06	N	N	N
49	Serum	33.99	N	N	N
50	Serum	33.12	N	N	N
51	Testicular Fluid	21.3	N	N	N
52	Testicular Fluid	17.69	N	N	N
53	Serum	34.9	N	N	N
54	Serum	34.39	N	N	N
55	Serum	33.75	N	N	N
56	Serum	33.24	N	N	N
57	Serum	32.85	N	N	N
58	Serum	31.46	N	N	N
59	Serum	29.92	N	N	N
60	Serum	37.61	N	N	N
61	Oral and Nasal swabs	26.79	N	N	N
62	Serum	32.81	N	N	N
63	Serum	17.37	29.01	N	N
64	Serum	16.52	27.73	N	N
65	Serum	15.95	26.87	N	N
66	Serum	15.97	27.2	N	N
67	Serum	17	28.54	N	N
68	Serum	21.96	N	N	N
69	Serum	21.43	42.46	N	N

70	Serum	34.95	N	N	N
71	Serum	27.98	N	N	N
72	Serum	22.97	N	N	N
73	Serum	26.62	N	N	N
74	Serum	33.83	N	N	N
75	Serum	16.91	27.93	N	N
76	Serum	15.36	25.86	N	N
77	Serum	16.18	26.81	N	N
78	Serum	19.1	30.4	N	N
79	Serum	16.05	26.85	N	N
80	Serum	19.19	30.25	N	N
81	Serum	17.74	36.16	N	N
82	Testicular Fluid	27.55	N	N	N
83	Testicular Fluid	21.91	N	N	N
84	Testicular Fluid	19.27	N	N	N
85	Testicular Fluid	29.92	N	N	N
86	Testicular Fluid	26.63	N	N	N
87	Testicular Fluid	26.69	N	N	N
88	Serum	30.26	N	N	N
89	Testicular Fluid	21.51	N	N	N
90	Testicular Fluid	29.59	N	N	N
91	Testicular Fluid	18.56	N	N	N
92	Tissue	33.53	N	N	N
93	Tissue	37.56	N	N	N
94	Tissue	31.17	N	N	N
95	Serum	22.39	N	N	N
96	Serum	19.83	33.29	N	N
97	Serum	24.63	N	N	N
98	Tissue	34.29	N	N	N
99	Tissue	28.43	N	N	N
100	Tissue	28.44	N	N	N
101	Tissue	30.66	N	N	N
102	Tissue	32.92	N	N	N
103	Tissue	31.61	N	N	N
104	Tissue	26.53	N	N	N
105	Tissue	30.06	N	N	N
106	Tissue	31.67	N	N	N
107	Tissue	23.22	N	N	N
108	Tissue	33.64	N	N	N
109	Tissue	29.19	N	N	N
110	Tissue	31.57	N	N	N
111	Tissue	33.26	N	N	N
112	Tissue	28.77	N	N	N
113	Serum	25.58	N	N	N

114	Testicular Fluid	25	N	N	N
115	Testicular Fluid	19.42	N	N	N
116	Testicular Fluid	20.46	N	N	N
117	Tissue	25.28	N	N	N
118	Oral and Nasal swabs	28.67	N	N	N
119	Oral and Nasal swabs	32.7	N	N	N
120	Testicular Fluid	19.36	N	N	N
121	Serum	33.39	N	N	N
122	Serum	33.9	N	N	N
123	Serum	34.12	N	N	N
124	Serum	29.3	N	N	N
